# Machine learning algorithms for predicting COVID-19 mortality in Ethiopia

**DOI:** 10.1186/s12889-024-19196-0

**Published:** 2024-06-28

**Authors:** Melsew Setegn Alie, Yilkal Negesse, Kassa Kindie, Dereje Senay Merawi

**Affiliations:** 1 Department Public Health, School of Public Health, College of Medicine and Health Science, Mizan-Tepi University, Mizan-Aman, Ethiopia; 2 Department of Public Health, College of Medicine and Health Science, Debre Markos University, Gojjam, Ethiopia; 3 Department Nursing, College of Medicine and Health Science, Mizan-Tepi University, Mizan-Aman, Ethiopia; 4 Department of Information Technology, Faculty of Technology, Debre Tabor University, Gonder, Ethiopia

**Keywords:** SARS-CoV-2 Infections, COVID 19 Pandemic, COVID-19, Pandemic, COVID-19 Ethiopia

## Abstract

**Background:**

Coronavirus disease 2019 (COVID-19), a global public health crisis, continues to pose challenges despite preventive measures. The daily rise in COVID-19 cases is concerning, and the testing process is both time-consuming and costly. While several models have been created to predict mortality in COVID-19 patients, only a few have shown sufficient accuracy. Machine learning algorithms offer a promising approach to data-driven prediction of clinical outcomes, surpassing traditional statistical modeling. Leveraging machine learning (ML) algorithms could potentially provide a solution for predicting mortality in hospitalized COVID-19 patients in Ethiopia. Therefore, the aim of this study is to develop and validate machine-learning models for accurately predicting mortality in COVID-19 hospitalized patients in Ethiopia.

**Methods:**

Our study involved analyzing electronic medical records of COVID-19 patients who were admitted to public hospitals in Ethiopia. Specifically, we developed seven different machine learning models to predict COVID-19 patient mortality. These models included J48 decision tree, random forest (RF), k-nearest neighborhood (k-NN), multi-layer perceptron (MLP), Naïve Bayes (NB), eXtreme gradient boosting (XGBoost), and logistic regression (LR). We then compared the performance of these models using data from a cohort of 696 patients through statistical analysis. To evaluate the effectiveness of the models, we utilized metrics derived from the confusion matrix such as sensitivity, specificity, precision, and receiver operating characteristic (ROC).

**Results:**

The study included a total of 696 patients, with a higher number of females (440 patients, accounting for 63.2%) compared to males. The median age of the participants was 35.0 years old, with an interquartile range of 18–79. After conducting different feature selection procedures, 23 features were examined, and identified as predictors of mortality, and it was determined that gender, Intensive care unit (ICU) admission, and alcohol drinking/addiction were the top three predictors of COVID-19 mortality. On the other hand, loss of smell, loss of taste, and hypertension were identified as the three lowest predictors of COVID-19 mortality. The experimental results revealed that the k-nearest neighbor (k-NN) algorithm outperformed than other machine learning algorithms, achieving an accuracy of 95.25%, sensitivity of 95.30%, precision of 92.7%, specificity of 93.30%, F1 score 93.98% and a receiver operating characteristic (ROC) score of 96.90%. These findings highlight the effectiveness of the k-NN algorithm in predicting COVID-19 outcomes based on the selected features.

**Conclusion:**

Our study has developed an innovative model that utilizes hospital data to accurately predict the mortality risk of COVID-19 patients. The main objective of this model is to prioritize early treatment for high-risk patients and optimize strained healthcare systems during the ongoing pandemic. By integrating machine learning with comprehensive hospital databases, our model effectively classifies patients' mortality risk, enabling targeted medical interventions and improved resource management.

Among the various methods tested, the K-nearest neighbors (KNN) algorithm demonstrated the highest accuracy, allowing for early identification of high-risk patients. Through KNN feature identification, we identified 23 predictors that significantly contribute to predicting COVID-19 mortality. The top five predictors are gender (female), intensive care unit (ICU) admission, alcohol drinking, smoking, and symptoms of headache and chills.

This advancement holds great promise in enhancing healthcare outcomes and decision-making during the pandemic. By providing services and prioritizing patients based on the identified predictors, healthcare facilities and providers can improve the chances of survival for individuals. This model provides valuable insights that can guide healthcare professionals in allocating resources and delivering appropriate care to those at highest risk.

**Supplementary Information:**

The online version contains supplementary material available at 10.1186/s12889-024-19196-0.

## Background

Coronavirus disease-2019 (COVID-19), a global health emergency declared by the World Health Organization (WHO) in January 2020, has rapidly spread worldwide, resulting in millions of infections and hundreds of thousands of deaths [1, 2]. Among African countries, Ethiopia is one of the most affected by the pandemic [3]. As of March 12, 2024, the global prevalence of COVID-19 reached 704,000,253 confirmed cases, with a total of 7,004,732 deaths reported [4]. In Ethiopia, there were 501,117 confirmed cases, out of which 488,171 individuals have successfully recovered. Unfortunately, the country also recorded 7,574 deaths due to COVID-19. These numbers highlight the ongoing challenges posed by the pandemic and the importance of adhering to preventive measures to mitigate its impact [5].

The clinical outcomes of the virus range from asymptomatic or mild symptoms to severe complications and, in some cases, even death. Coronavirus disease 2019 (COVID-19) is a highly contagious viral infection that continues to spread rapidly worldwide, posing a significant global health concern. The rapid spread of the virus has led to a severe shortage of medical resources and the exhaustion of frontline healthcare workers [6–10]. Additionally, many COVID-19 patients experience a rapid deterioration in their condition after initially experiencing mild symptoms, highlighting the need for advanced risk stratification models. By employing predictive models, it is possible to identify patients who are at an increased risk of mortality and provide them with timely support to reduce the number of deaths [11–15]. This is crucial in order to alleviate the burden on the healthcare system and ensure the best possible care for patients. Given the uncertainty surrounding the disease's ultimate impact, clinicians and health policymakers often rely on predictions generated by various computational and statistical models. These predictions help inform decision-making and guide interventions to effectively triage critically ill patients and improve the survivor [16, 17].

Healthcare systems worldwide are facing various challenges, prompting them to explore the potential of machine learning (ML) classifiers as a means of making more objective decisions and reducing reliance on subjective evaluations by physicians [18, 19]. ML, a branch of artificial intelligence (AI), allows for the extraction of high-quality predictive models from vast datasets [19, 20]. In the field of medical research, ML is increasingly utilized to enhance predictive modeling and uncover new factors that contribute to specific outcomes [20, 21]. Physicians often struggle to accurately predict the prognosis of COVID-19 patients when they are admitted to the hospital. Even patients who appear stable can experience sudden and severe deterioration, making it difficult for even the most skilled doctors to anticipate their progression. To improve the accuracy of clinical predictions, AI models can be valuable tools, as they are capable of detecting complex patterns in large datasets that the human brain cannot easily discern [22–24]. AI has been employed on various fronts in the fight against COVID-19, from epidemiological modeling to individualized diagnosis and prognosis prediction. While several COVID-19 prognostic models have been proposed, no comprehensive study has yet evaluated and compared the predictive power of non-invasive and invasive features [18, 25–28].

Several research studies [29–36] have been conducted to predict the mortality rate of patients with COVID-19 on a global scale. These studies have identified various significant factors that contribute to the prediction of mortality in COVID-19 patients. Different researchers were conducted different prediction model of machine learning and identified important features. Various studies have utilized different machine-learning algorithms to identify features that can be used to predict mortality in COVID-19 patients. These features include age [12, 17, 37–45], gender [11, 18, 28, 37, 39, 40, 43–46], dry cough [15, 17, 18, 28, 37, 40, 41, 43, 47], as the clinical symptom, underlying diseases including cardiovascular disease [37, 38, 40–42, 46, 48, 49], hypertension [37, 38, 41, 43, 44, 46, 50], diabetes [37–40], neurological disease [37, 39, 40], cancer [12, 37, 40, 43, 49]. Additionally laboratory indices such as serum creatinine [37, 40], RBC [37], WBC [15, 37, 43], hematocrit [37], absolute lymphocyte count [11, 28, 37, 40, 41, 46, 47], absolute neutrophil count [15, 17, 28, 37, 40–42, 47, 48], calcium [17, 28, 37], phosphor [37], blood urea nitrogen [28, 37, 47], total bilirubin [15, 37], serum albumin [28, 37, 43, 46], glucose [37, 40], creatinine kinase [15, 17, 37, 43, 46, 47], activated partial thromboplastic time [37], prothrombin time [37, 46], and hypersensitive troponin [37, 40, 42].

By providing evidence-based medicine for risk analysis, screening, prediction, and care plans, ML algorithms can reduce uncertainty and ambiguity, supporting reliable clinical decision-making and ultimately leading to improved patient outcomes and quality of care [51, 52]. In Ethiopia the some studies were conducted on COVID-19 mortality [53–57] while only one studies [58] were conducted to predict mortality using machine-learning algorithms. However, these studies did not take into account important factors such as demographic, clinical, and laboratory predictors of COVID-19 mortality. It has been observed that these features have a correlation with the mortality of individuals during hospitalization [40, 41, 45]. To address this gap, new non-invasive digital technologies, including machine-learning prediction have been introduced for predicting the mortality of COVID-19 patients. Machine-learning systems learn from past experiences and can adapt to new inputs, making them valuable tools in mortality prediction. Machine learning (ML), a subfield of AI, is a sophisticated and flexible classification modeling technique that utilizes large datasets to uncover hidden relationships or patterns [59]. Compared to conventional statistical models, ML methods have shown more accurate results in predicting clinical outcomes for COVID-19 patients. These ML-based models have been primarily evaluated using demographics, risk factors, clinical manifestations, and laboratory results to assess their prognostic performance [38–40, 47]. Incorporating demographic, clinical, and laboratory features into machine learning algorithms has shown promise in enhancing the accuracy of mortality prediction for COVID-19 patients, thereby improving patient care and outcomes during the ongoing pandemic. However, a gap in the existing literature was identified regarding the use of ensemble modeling and machine learning algorithms for predicting COVID-19 mortality in Ethiopia. Thus, the primary objective of the study was to compare the effectiveness of various machine learning techniques in predicting COVID-19 mortality in Ethiopia. The accurate prognosis of COVID-19 clinical outcome is challenging due to the wide range of illness severity, which makes appropriate triage and resource allocation crucial for enhancing patient care within health-care systems. The study introduces a novel machine learning ensemble algorithm specifically designed for predicting COVID-19 mortality in Ethiopia, showcasing the application of machine learning algorithms to daily recorded data, such as the daily mortality rate of COVID-19. Overall, the study provides valuable insights and technical contributions to the field of COVID-19 mortality prediction.

## Methods

### Patient selection

**Fig. 1:**
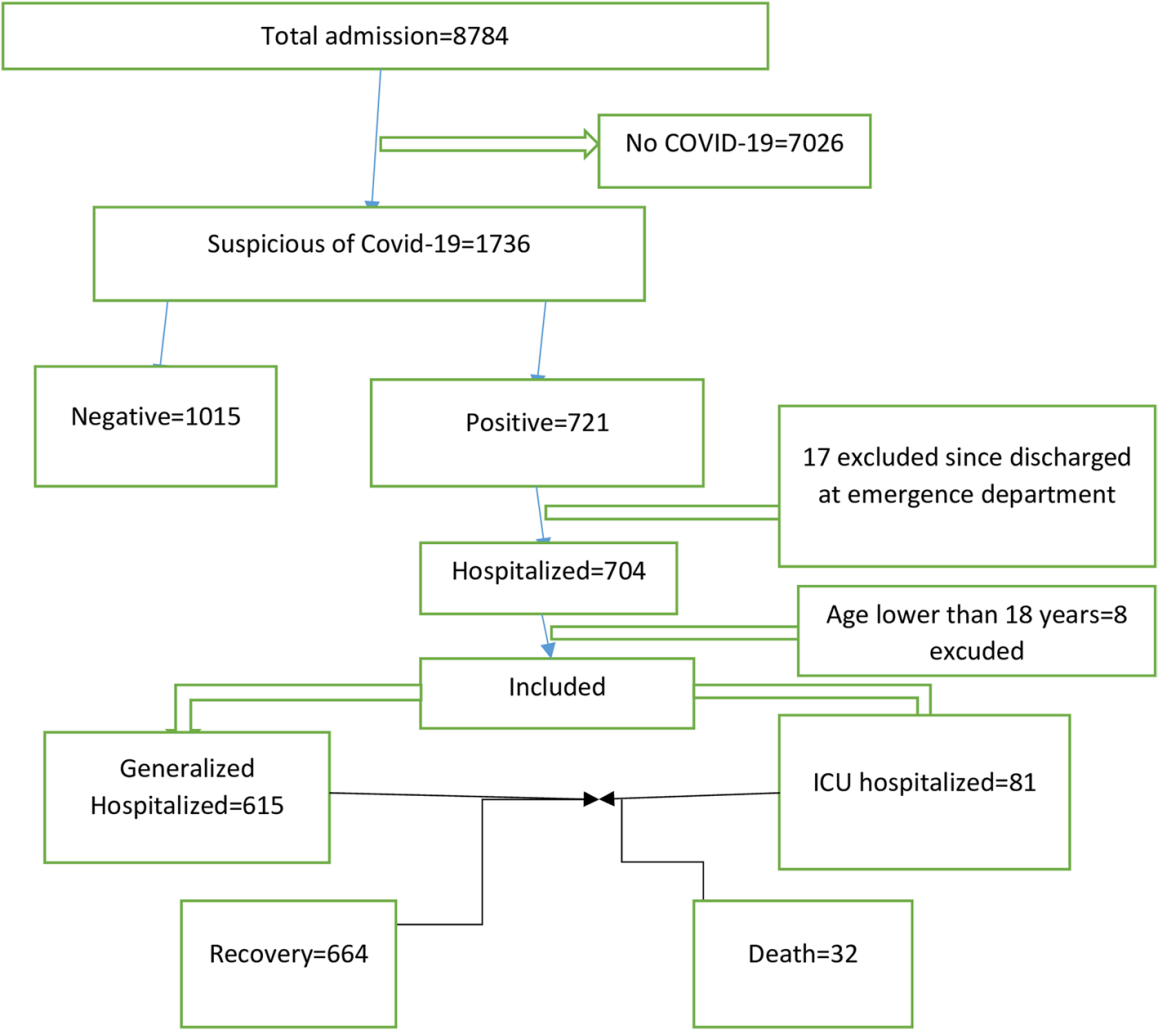
Flowchart of patient selection in the current study

The study utilized data from a database registry in Ethiopian hospitals, specifically from the district health information system report of the hospitals. Out of the total 8784 admissions, 7026 were excluded as they did not show any COVID-19 symptoms. From the remaining 1736 suspected cases, 1015 individuals were excluded as they tested negative for COVID-19. The analysis included a total of 696 hospitalized patients who were confirmed to have COVID-19, identified from the pool of suspected cases. The patients hospitalized from January 1-March 5, 2022 was retrospectively reviewed and included in this study. Confirmation was based on real-time polymerase chain reaction (RT-PCR) tests, which is a reliable method for diagnosing COVID-19. The study period ran from March 10, 2022, to May 10, 2022. To ensure data accuracy, two health information management experts thoroughly reviewed all the collected data. Moreover, physicians who completed the questionnaire and patients or their family members were contacted to review and supplement any missing data or clarify any differing interpretations. The detailed inclusion and exclusion criteria were presented in Fig. 1. The data obtained from hospitals were exported to Statistical Package for the Social Sciences (SPSS), Waikato Environment for Knowledge Analysis (WEKA) and R software for further for analysis. After exporting the SPSS file was further exported to WEKA, a widely used software tool for data mining and machine learning. The analysis was carried out using Weka, which helped to identify the most significant clinical and demographic features that could assist in predicting mortality in COVID-19 patients.

### Data source and dataset description

The data from hospital register of district health information system 2 excel data were obtained from the selected hospitals. In this study, the input features were identified based on the hospital registers of each patient. A COVID-19 hospital-based registry database was retrospectively reviewed from January 1, 2022, to March 5, 2022. The database included forty-six [46] primary features, categorized into five main classes: patient demographics, clinical features, risk factors, laboratory results, and an output variable indicating survival (0: survived and 1: deceased). Numerical parameters were quantitatively measured, while nominal parameters were registered as "Yes" or "No". Demographic information and the risk factors were obtained from the medical records of the patients. Clinical features such as cough, fever, shortness of breath, loss of smell, loss of taste, and others were registered at the time of admission. Within the first 24 h of hospitalization, blood and urine samples were analyzed, and the laboratory results were automatically recorded in the patients' medical records. To ensure data quality, a two-round Delphi survey was conducted to address noisy and abnormal values, errors, and meaningless data. The Excel file from district health information system (DHS2) were exported to SPSS, Waikato Environment for Knowledge Analysis (WEKA) and R software for further analysis. The analysis was done by SPSS and WEKA software. WEKA software were used for data mining of machine learning algorithms. The data analyzed for this study was obtained from reasonable request from crossponding author.

### Outcome variable

In this study, the outcome variable was defined as "deceased," which indicated whether a patient had experienced in-hospital mortality due to COVID-19. The variable was represented using a binary distribution, with "Yes" indicating that the patient had passed away and "No" indicating that they recovered from COVID-19.

### Data preprocessing

In this specific study, we made the decision to exclude patients who were below 18 years of age and those who were discharged from the emergency department with unknown outcomes. Our data was obtained from a de-identified hospital registry database, which consisted of information from a total of 696 patients (as depicted in Fig. 1). To ensure the quality of our data, a collaborative effort was made by two health information management experts, along with two epidemiologists and hematology specialists. Their expertise was utilized to identify and address any noisy or abnormal values, errors, duplicates, and meaningless data. Additionally, we reviewed the initial list of parameters to ensure consistency in the preprocessing of the data.

Ultimately, our analysis focused on data from 696 hospitalized patients who were 18 years old or older. For a more comprehensive understanding of the exclusion criteria applied in the study, please refer to Fig. 1, which provides a visual representation of the study's inclusion criteria.

### Data balancing

Imbalanced data poses a significant challenge in machine learning algorithms, where the distribution of classes in a dataset is uneven. In the current dataset being analyzed, there is a substantial imbalance between the "alive" and "death" classes, with 664 and 32 cases, respectively. This imbalance can lead to inaccurate results and make it likely for new observations to be categorized into the majority class. To address this issue, the study employed a technique called Synthetic Minority Over-sampling Technique (SMOTE) from the imbalanced-learn toolbox. SMOTE generates synthetic samples for the minority class by interpolating between existing minority class samples. By applying SMOTE, the dataset was balanced, allowing for more accurate and unbiased training of machine learning models. If you are interested in learning more about the imbalanced-learn toolbox and the SMOTE method, you can visit their website at https://imbalanced-learn.org/stable/. While predictive accuracy is a commonly used metric to evaluate the performance of machine learning algorithms, it can be misleading when working with imbalanced datasets. In this particular study, researchers employed various techniques to address the class imbalance issue in their dataset. They utilized Synthetic Minority Oversampling Technique (SMOTE) [60], which generates new samples by interpolating between existing samples and their neighbors [61, 62]. Additionally, they employed random under-sampling, which involves discarding samples from the majority class until the minority class reaches a predetermined percentage of the majority class [60]. Another method used was Adaptive Synthetic (ADASYN), which generates synthetic data for harder-to-learn minority class samples, thereby reducing bias introduced by imbalanced data distribution. Through the application of these techniques, the researchers successfully achieved a balanced dataset [63]. The outcome of their endeavors is discussed in detail in the result section of the study.

### Feature selection and methods

In the initial phase of our study, our primary aim was to identify key clinical features that could effectively predict mortality in COVID-19 patients. To achieve this, we conducted an extensive review of scientific literature by searching various databases. The findings from this review were then utilized to create a comprehensive questionnaire, which encompassed a wide range of predictors, including patient demographics, risk factors, clinical manifestations, and laboratory tests. To ensure the validity of the questionnaire, we assembled a panel of experts consisting of two epidemiologists and two laboratory assistant professors. These experts meticulously assessed the content and provided valuable input based on their expertise. Through a combination of the literature review and the panel's discussions, we were able to determine a finalized set of features. To evaluate the importance of each feature, we reviewed the initial list of parameters and scored each item based on its predictive value for COVID-19 mortality. The scoring was conducted using a 5-point Likert scale, ranging from 1 (not important) to 5 (highly important). Only the features with an average score of 3.75 (70%) or higher were considered for inclusion in the study [40]. The results of the Delphi-discussion, which incorporated the findings from the panel's deliberations, are presented in supplementary Table 1 of the revised manuscript. These results were also incorporated into the manuscript as Supplementary file 1. This set of features was then utilized to collect the necessary data for our study. By incorporating these identified predictors, our objective was to develop a reliable tool for predicting mortality in COVID-19 patients. Additionally, the admission time data were also incorporated to enhance the presentation of our data.

**Fig. 2:**
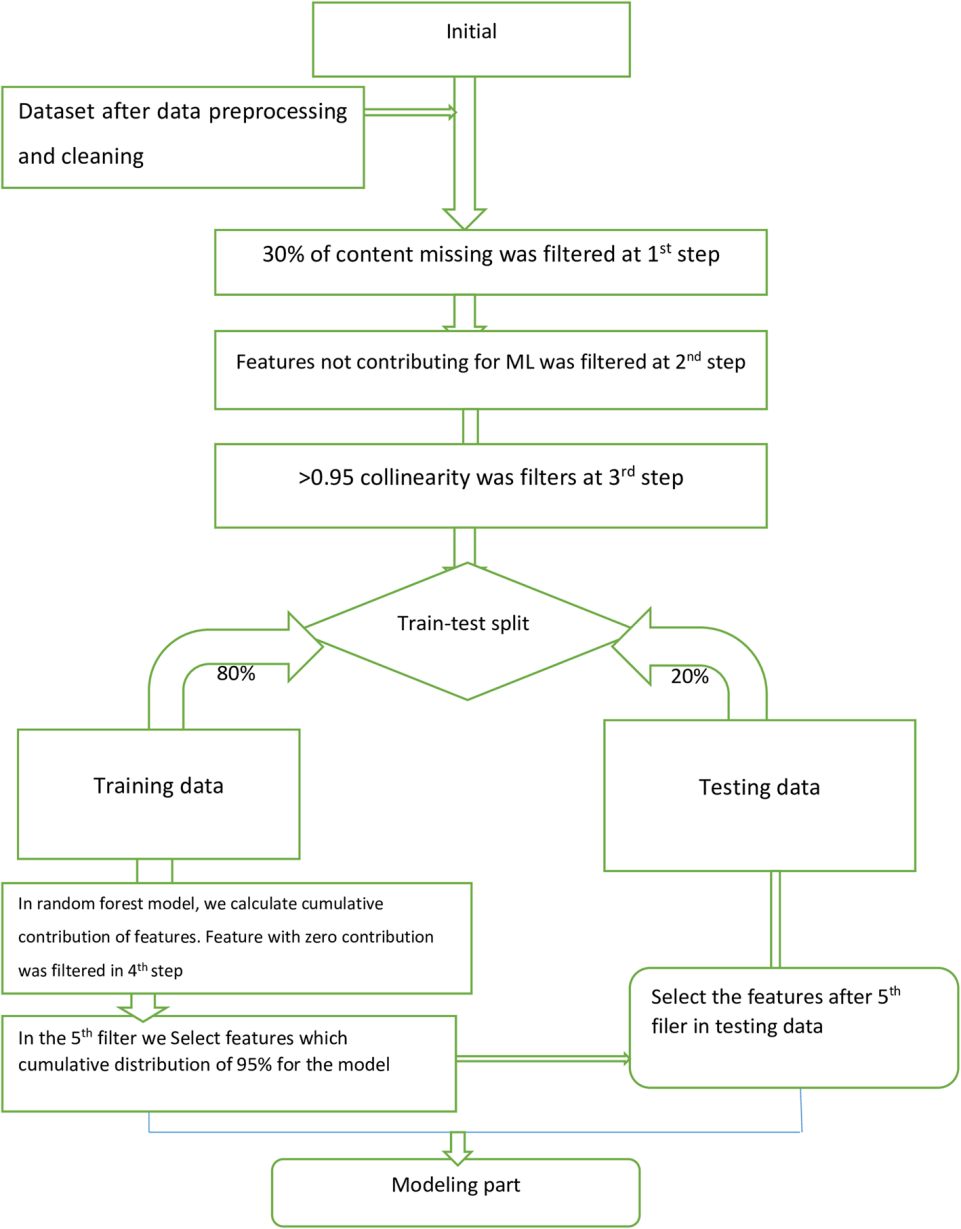
The flowchart of variable selection for machine-learning algorithm model

In Fig. 2, you can observe the flowchart outlining the feature selection process, which involved five distinct steps to select the final variables. The first step involved removing features that had a missing value greater than 30% from the dataset. In the second step, we focused on eliminating features that did not significantly contribute to the machine-learning model, such as reference date, patient ID, and accompanying information, as these were deemed irrelevant to our final outcome variable. The third step aimed to address collinearity, which can result in duplicated features and skew the model's results. Features with a collinearity greater than 0.95 were eliminated from the dataset. By implementing these procedures, we were able to identify the most relevant and informative features for our machine learning model (see Fig. 2). In this study, several feature selection methods were utilized to determine the most relevant predictive features. These methods included recursive feature elimination (RFE), correlation coefficient, random forest feature importance, and the Boruta feature selection method. RFE is a technique employed for feature selection, which starts with all the features in the training dataset and gradually eliminates features until the desired number is achieved. This method is particularly effective in reducing model complexity and enhancing the efficiency of machine learning algorithms. By employing these feature selection methods, the study aimed to identify the most informative features that significantly contribute to the predictive power of the model [64]. This approach streamlines data processing and improves the accuracy of machine learning algorithms.

### Model development

A comprehensive literature review was conducted to develop accurate predictive classifier models for COVID-19 mortality. The review included studies referenced as [11, 12, 15, 18, 28, 38, 39, 41, 46, 51, 65, 66]. The selection of suitable machine learning (ML) algorithms was based on the type and quality of the dataset utilized. Seven ML algorithms were employed to construct the mortality prediction model: J48 decision tree, random forest (RF), k-nearest neighborhood (k-NN), multi-layer perceptron (MLP), Naïve Bayes (NB), eXtreme gradient boosting (XGBoost), and logistic regression (LR). The data was analyzed using WEKA software v3.9.2 was used to implement the algorithms, analyze and calculate curves and criteria, and draw the confusion matrix.

### Cross-validation

**Fig. 3:**
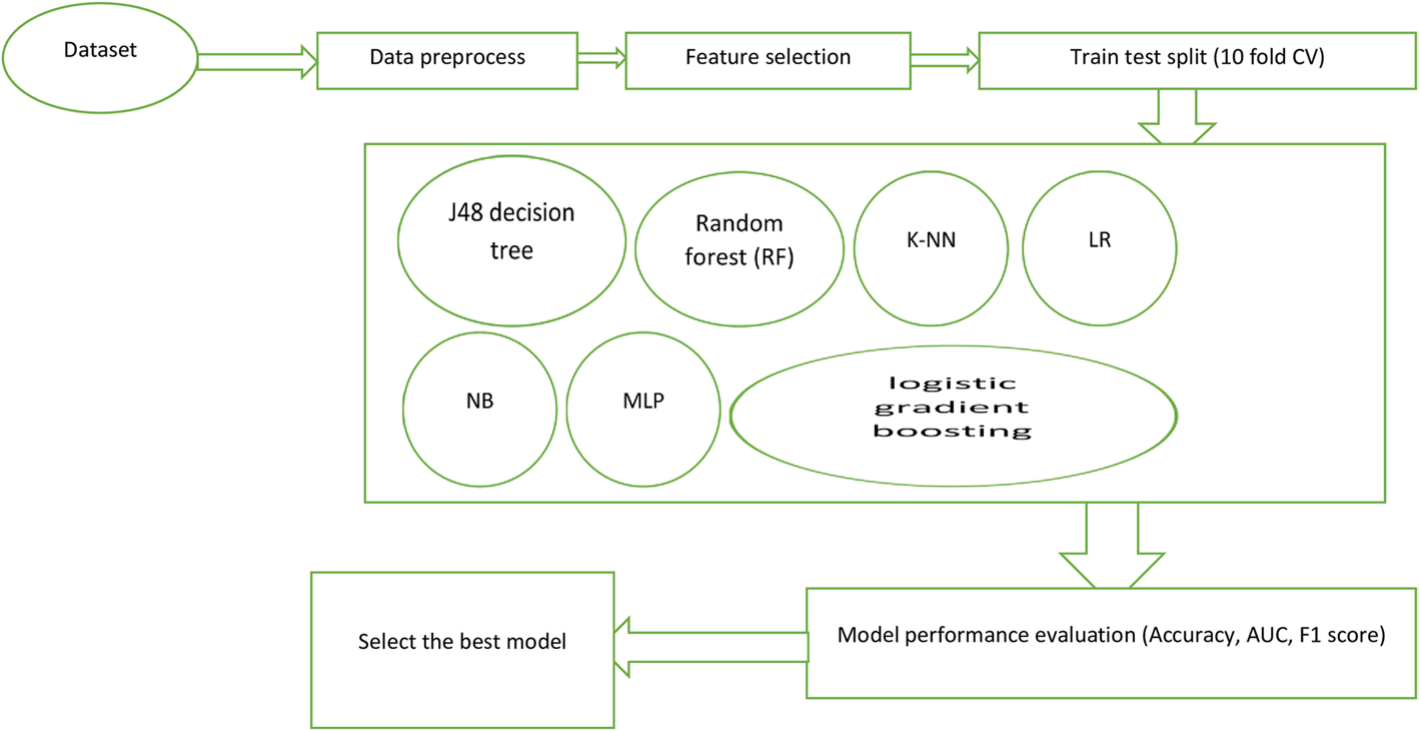
Workflow of machine learning for prediction of COVID-19 mortality

In our study, we utilized the EXPLORER module of WEKA to determine the optimal hyper parameters for the models we used. We selected the hyper parameters that achieved the best performance values. To evaluate the performance and general error of the classification models, we employed a tenfold cross-validation process. This process involved dividing the data into ten subsets, where one subset was used as the validation dataset and the remaining nine subsets were used as training datasets. We repeated this process ten times, ensuring that each subset was used as the validation dataset once. This approach helped us obtain reliable performance metrics. To facilitate the comparison of predictive performance, we ran all models ten times using WEKA's EXPERIMENTER module and repeated the tenfold cross-validation. This ensured that the validation results were based on samples of approximately equal size. By combining the validation results from the ten experimental models, we obtained performance metrics such as sensitivity, specificity, accuracy, precision, and ROC derived from the testing phase. This approach allowed us to accurately assess and compare the performance of the models. Furthermore, we have calculated the average performance metrics across the five runs to provide a more comprehensive evaluation. We have chosen to use stratified fivefold cross-validation as it strikes a favorable balance between bias and variance, making it a preferred technique for accurately estimating accuracy. It is worth emphasizing that tenfold cross-validation is widely employed in the fields of machine learning and data mining due to its advantages over traditional instance splitting methods. This approach helps minimize deviations in prediction errors, allowing for the utilization of more data for both training and validation purposes without the risk of overfitting or overlap. Additionally, it safeguards against biases that may arise from arbitrary data splitting. By utilizing the EXPLORER and EXPERIMENTER modules in WEKA, in conjunction with fivefold cross-validation, our approach provides a robust and reliable method for assessing and comparing the effectiveness of classification models. The flowchart of machine- learning prediction clearly put in Fig. 3.

### Model evaluation

Evaluating the performance of a machine learning model is crucial for its success. In our study, we assessed the performance of our predictive models using a range of performance metrics, as outlined in Table 1. These metrics included accuracy, specificity, precision, sensitivity, and the receiver operating characteristic (ROC) chart criteria. By utilizing these metrics, we were able to effectively measure the effectiveness of our models in predicting COVID-19 mortalities. To identify the best model for predicting COVID-19 mortalities, we compared the performance of each model using the aforementioned evaluation criteria. The results of this comparison are summarized in Table 2. Through a careful analysis and comparison of these evaluation criteria, we were able to identify the model that demonstrated the highest performance in predicting COVID-19 mortalities. Our comprehensive evaluation process allowed us to select the most effective model and gain valuable insights and confidence in its predictive capabilities.

**Table 1:** Confusion matrix

Output	Predicted value	
Actual values	Death(+)	Survivor(-)
Death( +)	True positive (TP)	False negative (FN)
Survival(-)	False positive (FP)	True Negative (TN)

**Table 2:** The performance evaluation measures

Performance criteria	Calculation
Accuracy	$\frac{TP+TN}{TP+TN+FP+FN}$
Sensitivity/recall	$\frac{TP}{TP+FP}$
Precision	$\frac{TP}{TP+FN}$
Specificity	$\frac{TN}{TN+FP}$
F1-Secor	$$\frac{2*precision*recall}{precision+recall}$$

### Mathematical modelling

Random forest is a powerful ensemble learning algorithm that works by creating multiple decision trees, with the final output being determined by a voting process [30, 67]. This approach greatly reduces the impact of noise and outliers compared to using a single decision tree [29]. However, due to the complexity of the computation involved, even a small change in the input data can result in a different output. Despite this limitation, random forest remains a popular and effective tool for a variety of machine learning tasks [29, 68]. Random Forest (RF) ensembles consist of multiple decision trees, each constructed using a bootstrapped random sample of the available data. During the construction process, only a random selection of features is considered at each splitting node. To classify a new observation using the RF model, each decision tree in the ensemble "votes" for the class it predicts. The class that receives the majority vote from the decision trees is then considered as the prediction of the RF classifier. This reliance on the majority vote for classification allows the RF model to achieve better performance compared to a single decision tree classifier. The mathematical modeling and formula for each sections of random forest presented on Table 3

**Table 3:** The mathematical formulas for random forest

Impurity	Task	Formula	Description
Gini	Classification	$\sum\limits_{i=1}^{C}fi(1-fi)$	Fi = frequency of I labels at a node, C = number of unique labels
Entropy	Classification	$\sum\limits_{i=1}^{c}-filog(fi)$	Fi = frequency of I labels at a node, C = number of unique labels
Mean squared error (MSE)	Regression	$\frac{1}{N}\sum\limits_{I=1}^{N}\left(fi-\mu \right)2$	Yi = label for instances, *N* = the number of instances, $\mu$ =The mean given by = $\frac{1}{N}\sum\nolimits_{I=1}^{N}yi$
Mean absolute error (MAE)	Regression	$\frac{1}{N}\sum\limits_{I=1}^{N}|fi-\mu |$	Yi = label for instances, *N* = the number of instances, $\mu$ =The mean given by = $\frac{1}{N}\sum\nolimits_{I=1}^{N}yi$

Logistic regression is a statistical technique that utilizes the sigmoid function as its fundamental method. It is commonly employed in machine learning to construct models when the target variable is binary, such as determining whether a patient is deceased or alive [13, 69]. This algorithm is renowned for its simplicity in implementation, interpretation, and training. However, it tends to overfit when dealing with high-dimensional data and may struggle to capture intricate relationships [70]. The mathematical equation for logistic regression is as follows:

$$\mathrm{Decision}\;\left(\mathrm x\right)=\left\{\begin{array}{ll}1&if\;P\left(y=1\vert x\right)>0.5\\0&otherwise\end{array}\right\}$$

Where: X is an instance and P(y = 1|x) = the probability, 0.5 = Decision boundary.

Naive Bayes is a classification algorithm that can be used for both binary and multi-class classification tasks [71, 72]. It is based on the Bayes theorem and uses statistical methods to predict the probability of a given sample belonging to a particular class. One of the key advantages of this algorithm is its ability to handle large databases with high speed and robust performance [73–75].

$$\text{P}(\text{Y}|\text{X})=\frac{P\left(X|Y\right)*P(Y)}{P(X)}\text{---------------------Bayes theory}$$

Where P(X|Y) = posterior probability P(y | X) from the likelihood P(X | y), P(Y) = prior probabilities P(y) and P(X) = prior probabilities P(X) [76]

$$Y={\mathrm{argmaX}}_{\mathrm Y}\mathrm P\left(\mathrm Y\right)\prod\nolimits_{i=1}^nPxi\vert y\text{----------Naive Bayes classifier}$$

Where

xj: represents a feature/input variable (j) included in the model.

n: represents the total number of features in the data set.

p (yi): is the prior probability of the class/output variable.

p(xj|yi): is the likelihood of the feature, given the class variable yi.

MLP, or Multilayer Perceptron, is a popular feed-forward neural network algorithm comprising interconnected neurons that exchange information with each other [77, 78]. During training, each connection between the neurons is assigned a weight, which is adjusted to enable accurate output prediction [79]. The strength of MLP lies in its simplicity and effectiveness in handling datasets of various sizes. However, it should be noted that the computations involved in MLP can be complex and time-consuming [80]. The mathematical modeling for MLP α = $\Phi (\sum_{j}wjxj+b$

Where:- xj = inputs to the unit, the wj = weights, b = bias,

φ = none-linear activation function, and a = unit’s activation.

J48 decision tree is supervised machine learning algorithm employed for regression and classification tasks. It adopts a hierarchical structure resembling a tree, comprising a root node, branches, internal nodes, and leaf nodes [81, 82]. The primary objective of this algorithm is to reveal the underlying structural patterns inherent in the data. Notably, decision trees offer several advantages, including their speed, user-friendliness, and ability to handle high-dimensional datasets [79]. The mathematical equation for entropy and Gini index for decision trees shown below.

$$\text{Info}\left(\text{D}\right)=\sum\nolimits_{i=1}^{M}Pi({log}_{2}(pi),\text{Info}_{\text{A}}\left(\text{B}\right)=\sum\nolimits_{I=1}^{V}\frac{\left|Dl\right|}{\left|D\right|}*\text{Info}\left(\text{Dl}\right),\text{Gain}\left(\text{A}\right)=\text{ Info}\left(\text{D}\right)-\text{Info}_{\text{A}}\left(\text{D}\right), \text{SplitInfo}(\text{A})=\sum\nolimits_{i=1}^{v}\frac{|Dl|}{D}*{Log}_{2}\frac{|Dl|}{D}, \text{GainRatio}(\text{A})=\frac{Gain(A)}{SplitInfo(A)}$$

Where,

(Entropy) (Info(D))**:** It refers to the amount of information needed to classify a tuple in the dataset (D). It measures the uncertainty or randomness in the distribution of class labels within the dataset.

Probability (pi): It represents the likelihood that a randomly selected tuple in the dataset (D) belongs to a specific class (yi). The probability is calculated by dividing the number of tuples belonging to class yi by the total number of tuples in the dataset.

Information Needed after Splitting (InfoA(D)): This term quantifies the amount of information required to classify the tuples after using a specific feature (A) to split the dataset (D) into multiple partitions (v). Each partition corresponds to a mutually exclusive value (l) of the feature (A).

Information Gain (Gain(A)): It is the reduction in entropy or uncertainty achieved by partitioning the dataset based on a particular attribute (A). The higher the information gain, the more effective the attribute is in splitting the dataset and improving the classification accuracy.

Split Information (SplitInfo(A)): It is a normalization factor that takes into account the number of mutually exclusive values of an attribute (A). It is used to adjust the information gain by considering the potential bias introduced by attributes with a large number of values.

Gain Ratio (GainRatio(A)): It is a metric used in decision tree algorithms to evaluate the usefulness of each attribute during the tree generation process. It helps in selecting the most informative attribute for splitting the dataset.

The k-nearest neighbor algorithm is that can be applied to both classification and regression tasks. It leverages the concept of proximity to classify or predict the grouping of individual data points [83, 84]. This algorithm is known for its simplicity and ease of use, making it accessible even to those new to machine learning. However, it's important to note that k-nearest neighbor does come with certain drawbacks. One such drawback is its high computational cost, which means it may take longer to process large datasets. Additionally, the algorithm is sensitive to the structure of the data, meaning that the arrangement and distribution of the data points can significantly impact its performance. Lastly, k-nearest neighbor requires a relatively large storage space to store the entire training dataset, which can be a consideration when working with limited resources [79]. The mathematical equations for KNN classification and regression presented in the following formula [85]:

$$\mathrm{KNN}\;\mathrm{classification}=\left[{\mathrm C}_{q=mode\;\left\{Cni,Cn2,Cn3\dots.Cnk\right\}}\right]\;\mathrm{and}\;\mathrm{KNN}\;\mathrm{Regression}=\left[V_{q=\frac IK{\textstyle\sum_{i=1}^KV_{ni}}}\right]$$

$$\text{Similarity}(\text{x},\text{y})=-\sqrt{\sum\nolimits_{i=1}^{n}f(xi,yi)}=)=-\sqrt{\sum\nolimits_{i=1}^{n}(xi-yi)}2$$

Where,

xi: is the value of ith feature of observation x

yi: is the value of ith feature of observation y and

n: is the total number of features.

XGBoost, short for extreme gradient boosting algorithm, is a powerful ensemble learning algorithm known for its speed, user-friendly interface, and exceptional performance on large datasets [84, 86]. In XGBoost, decision trees are constructed sequentially, with each independent variable assigned a weight that serves as input for the decision tree. The weights are adjusted based on the prediction outcome and then fed into the next decision tree. This iterative process of ensemble prediction leads to a highly accurate and robust model [87]. The A-XGBoost algorithm is implemented by selecting columns from 1 to k as the input features, and column (k + 1) as the output variable in R. This is represented in the equation below.

$$\text{R}= \left(\begin{array}{c}{r}_{1} {r}_{2}\dots {r}_{k} {r}_{k+1}\\ {r}_{2} {r}_{3}\dots {r}_{k+1} {r}_{k+2}\\ .\quad .\quad.\quad. \quad\\ .\quad.\quad.\quad.\quad \\ .\quad .\quad .\quad . \quad\\ {r}_{n-k-1} {r}_{n-k}\dots {r}_{n-2} {r}_{n-1}\\ {r}_{n-k} {r}_{n-k+1}\dots {r}_{n-1} {r}_{n}\end{array}\right)(\text{n}-\text{k})*(\text{k}+1)$$

### Association rule mining

Association rule mining is a technique that explores correlations among multiple variables within a group. It was initially developed by Agarwal and Srikanth [88]. In a recent study, this technique was employed alongside the apriori algorithm to support the classification of machine learning algorithms for predicting COVID-19 mortality using R software [89]. By setting a minimal support degree of 0.00095 and a minimum confidence threshold of 90%, the researchers aimed to identify all potential association rules. A rule is considered reliable if its confidence level exceeds 80% [90]. The primary focus of this study was to identify features associated with adolescent HIV testing using association rules. Specifically, the researchers utilized a technique called classification association rules [91]. This involved analyzing the features implied by the target features (Antecedent = > Consequent). The ultimate goal was to classify the variables contributing to HIV testing among adolescents and identify the predictors associated with each category of testing. To evaluate the strength of each rule, the study employed metrics such as Support, Confidence, and Lift. It is worth noting that in this context, the feature sets represented by X and Y are mutually exclusive.

$$\begin{array}{l}\mathrm{Rule}\;\mathrm X=>\mathrm Y\\\mathrm{Support}=\frac{\text{Fequence}(\text{X},\text{Y})}{\mathrm N},\;\text{Confidence}=\frac{\text{Frequency}(\text{X},\text{Y})}{\text{Fequency}(\text{X})},\;\text{Lift}=\frac{\text{Frequency}(\text{X},\text{Y})}{\text{Fequency}(\text{X})\text{Frequency}(\text{Y})}\end{array}$$

## Results

### Patient characteristics and descriptive statistics

Following the application of our exclusion criteria and a quantitative analysis of case records, we have identified a total of 696 COVID-19 patients who were hospitalized and met the eligibility criteria for our study. Out of these participants, 63.2% or 440 patients were female, while 36.8% or 256 patients were male. The median age of the participants was 35.0 years old, with an interquartile range (IQR) of 19. A vast majority of the study participants, 91.5%, reported experiencing fever during their hospital admission. Additionally, 47.4% of the total 696 study participants required oxygen therapy during their hospitalization. For a more detailed overview of the qualitative and quantitative features analyzed in our study, please refer to Table 4 for the descriptive analysis of qualitative features and Table 5 for the descriptive analysis of quantitative features. These tables provide a comprehensive summary of the data collected and analyzed in our study.

**Table 4:** Descriptive statistics qualitative features of the current study

Feature(qualitative)	Value	Frequencies	Feature(qualitative)	Value	Frequencies
Gender	Male, female	256,440	Chest pain	Yes, no	58,638
Occupation	HW, Non employed	486,210	Shortness of breath	Yes, no	44,652
Cough	Yes, no	328,368	Hypertension	Yes, no	325,371
Confusion	Yes, no	343,353	Diabetes	Yes, no	345,351
Nausea/vomiting	Yes, no	230,466	Smoking	Yes, no	301,395
Headache	Yes, no	439,257	Alcohol drinking	Yes, no	264,432
Muscular pain	Yes, no	246,450	C-reactive protein	positive, negative	184,512
Chills	Yes, no	91,605	COPD	Yes, no	49,647
Fever	Yes, no	637,59	Chronic kidney disease	Yes, no	98,598
Pneumonia	Yes, no	117,579	Chronic liver disease	Yes, no	63,633
Oxygen therapy	Yes, no	330,366	Cancer	Yes, no	64,632
Dyspnea	Yes, no	281,415	Hematologic disease	Yes, no	45,651
Loss of smell	Yes, no	271,425	Malnutrition	Yes, no	67,629
Loss of taste	Yes, no	281,415	Tuberculosis	Yes, no	75,621
Runny noise	Yes, no	253,443	HIV/AIDS	Yes, no	63,633
other underline disease	Yes, no	206,490	ICU admission	Yes, no	335,361
Cardiac disease	Yes, no	222,474	Deceased	Recovered, died	664,32
Sore throat	Yes, no	69,627

**Table 5:** Descriptive statistics of quantitative the features of the current study

Features (quantitative)	Range	Median(IQR)
Age	18–79	35.0(19)
Body mass index	14.5–23.0	18.0(2.0)
Length of hospitalization	1–34 weeks	12.0(8)
White cell count	1350–345000	16,600(33,000)
platelet count	29,700–678000	457,000(199,000)
absolute lymphocyte count	12–96	34(10)
absolute neutrophil count	7–94	33(13)
Blood urea nitrogen	1–11	10(6)
Glucose	18–998	307(794)
lactate dehydrogenase	32.9–9996	515(117.75)
Alkaline phosphatase	9.2–2848	131(33)
Erythrocyte sedimentation rate	2–486	238(245.2)

### Feature selection

**Fig. 4:**
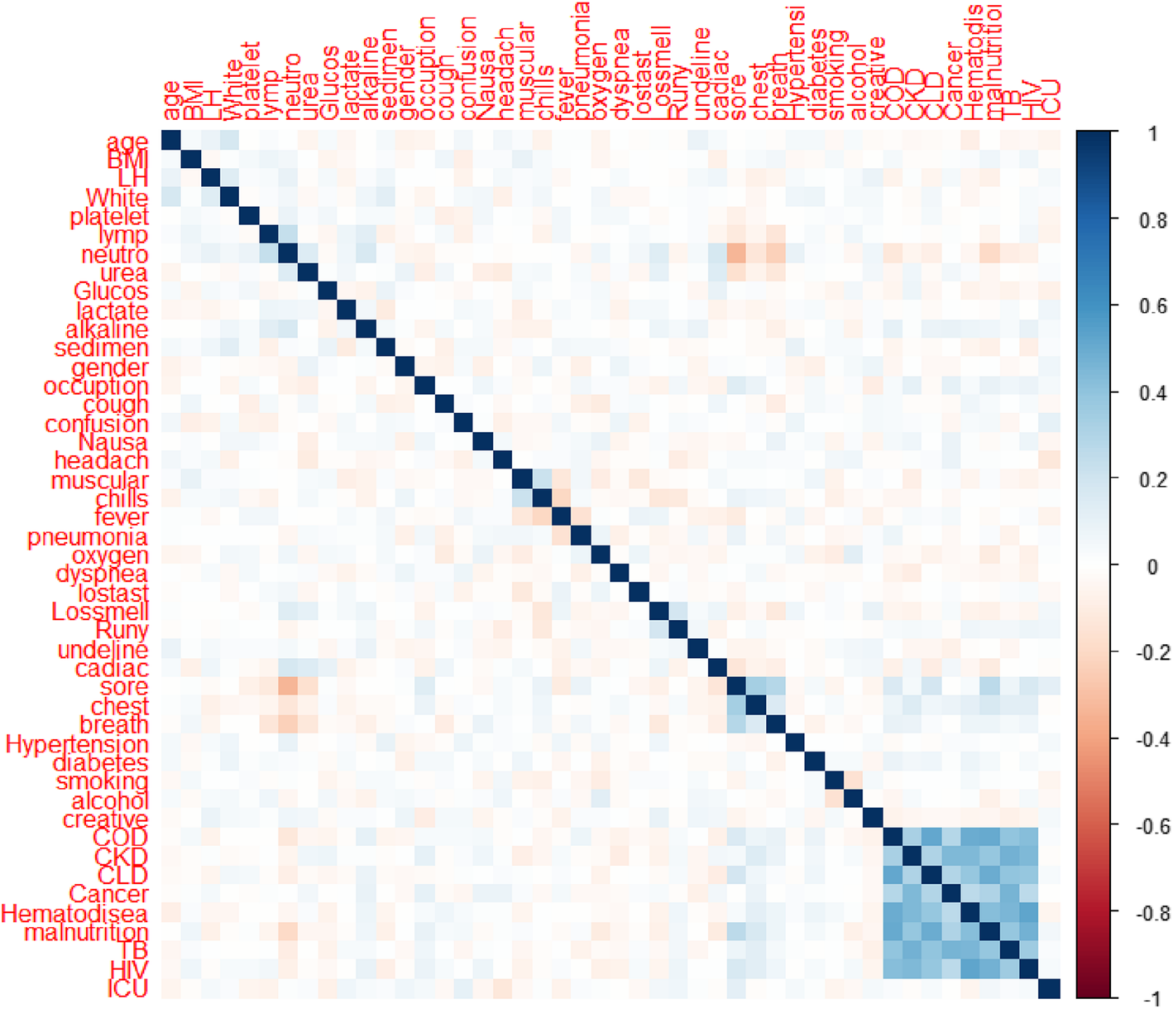
Correlation matrix of the feature for COVID-19 mortality in Ethiopia

**Fig. 5:**
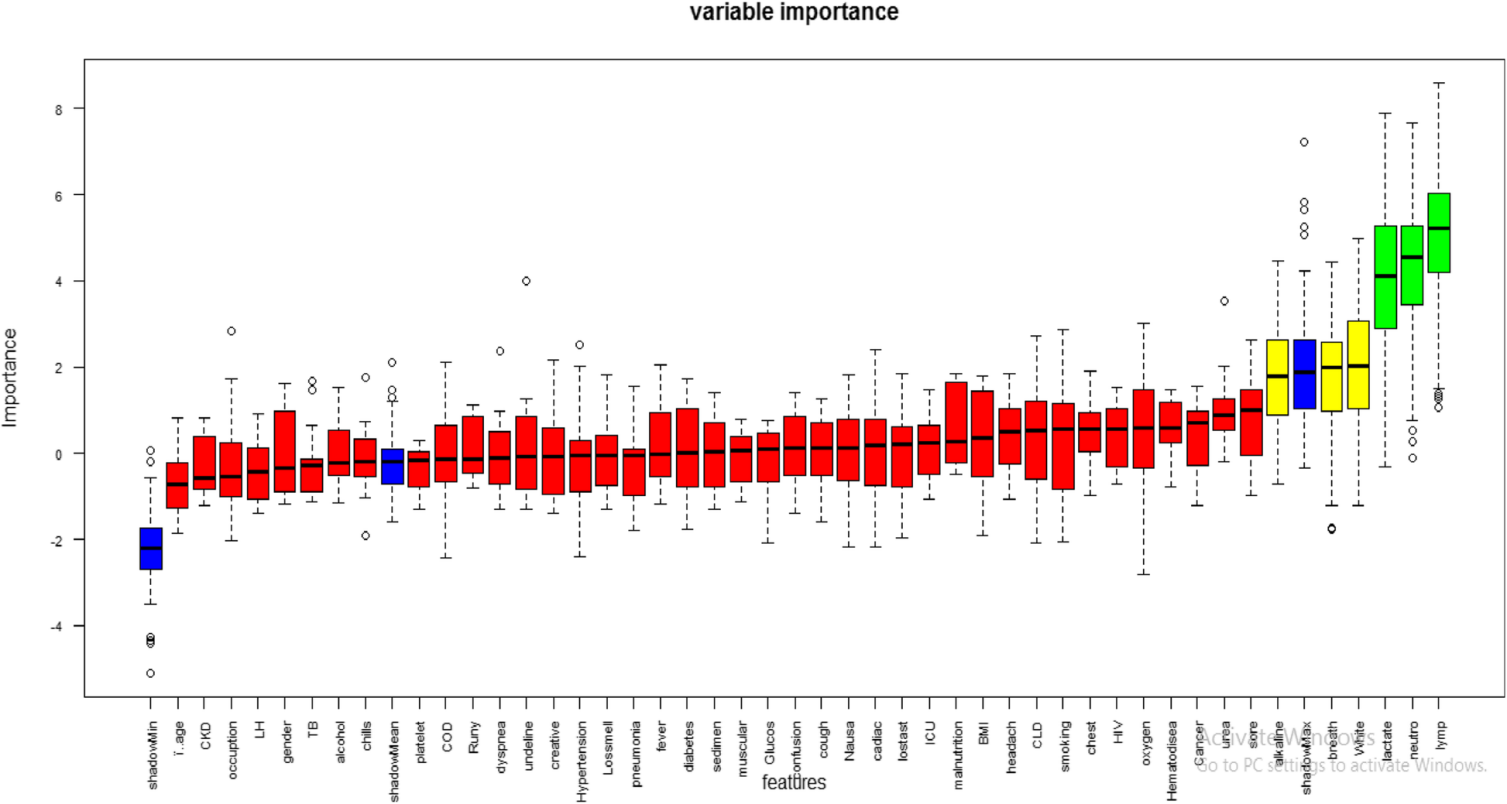
Feature selection by Boruta methods in COVID-19 mortality research

A thorough review of the literature has examined 46 factors that contribute to the risk of mortality from COVID-19. These factors were assessed for their significance using a feature evaluator, resulting in the identification of 23 features as highly important. However, 23 clinical and demographic features were included from the analysis and other feature were excluded based on specific criteria outlined in Fig. 2. The criteria were clearly specified in the Fig. 2 itself. To predict COVID-19 mortality among hospitalized patients, the significance of each factor was calculated, leading to the selection of 23 predictors for machine learning (ML) algorithms. These predictors were categorized into demographics, risk factors, clinical manifestations, laboratory tests, and therapeutic plans. Gender emerged as the most important predictor for COVID-19 mortality, with a value of 0.102857. On the other hand, hypertension was found to be the least important predictor, with a value of 0.01296. The importance of each feature in the dataset was calculated and presented in Table 6. The correlation matrix of the features were also presented in Fig. 4 which showed 23 variables were less correlated each other. The correlation matrix of the features clearly shown in Fig. 4. The Boruta feature selection also implemented in this machine-learning and presented on Fig. 5. The importance is from the left to right as showed in Fig. 5

**Table 6:** Features degree of importance in predicting mortality among patients with COVID‑19

S/n	Features name	Importance value	S/n	Features name	importance value
1	Gender	0.102857	12	Muscular pain	0.033155
2	ICU admission	0.060444	13	Chest pain	0.033095
3	Alcohol drinking	0.058505	14	Confusion	0.030598
4	Smoking	0.057618	15	Sore throat	0.026914
5	Headache	0.04526	16	Cardiac disease	0.026393
6	Chills	0.044441	17	Chronic liver disease	0.026383
7	Pneumonia	0.043648	18	Cough	0.026381
8	Oxygen therapy	0.043586	19	Hematologic disease	0.025973
9	Fever	0.042181	20	Nausea/vomiting	0.022966
10	TB	0.034331	21	Loss of smell	0.02167
11	COPD	0.033598	22	Loss of taste	0.015107
			23	Hypertension	0.01296

### Developing and evaluating models

We first choose the most optimal features for predicting COVID-19 mortality and then used seven different machine learning (ML) algorithms, namely J48, RF, LR, MLP, XGBoost, k-NN, and NB, to construct predictive models. To evaluate the performance of each algorithm, we conducted tenfold cross-validation with a seed value of two. We assessed the performance of each algorithm using various metrics, including sensitivity, specificity, accuracy, precision, and the receiver operating characteristic (ROC) curve. The results of the cross-validation are presented in Table 6.

**Fig. 6:**
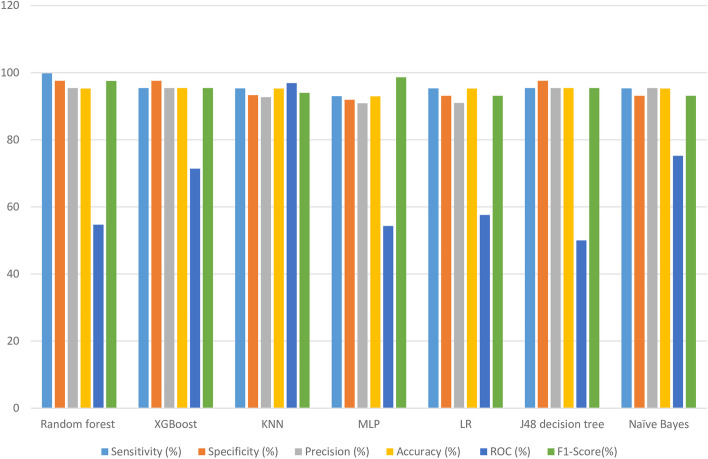
Visual comparisons of ML algorithm capabilities for COVID‑19 death prediction

**Fig. 7:**
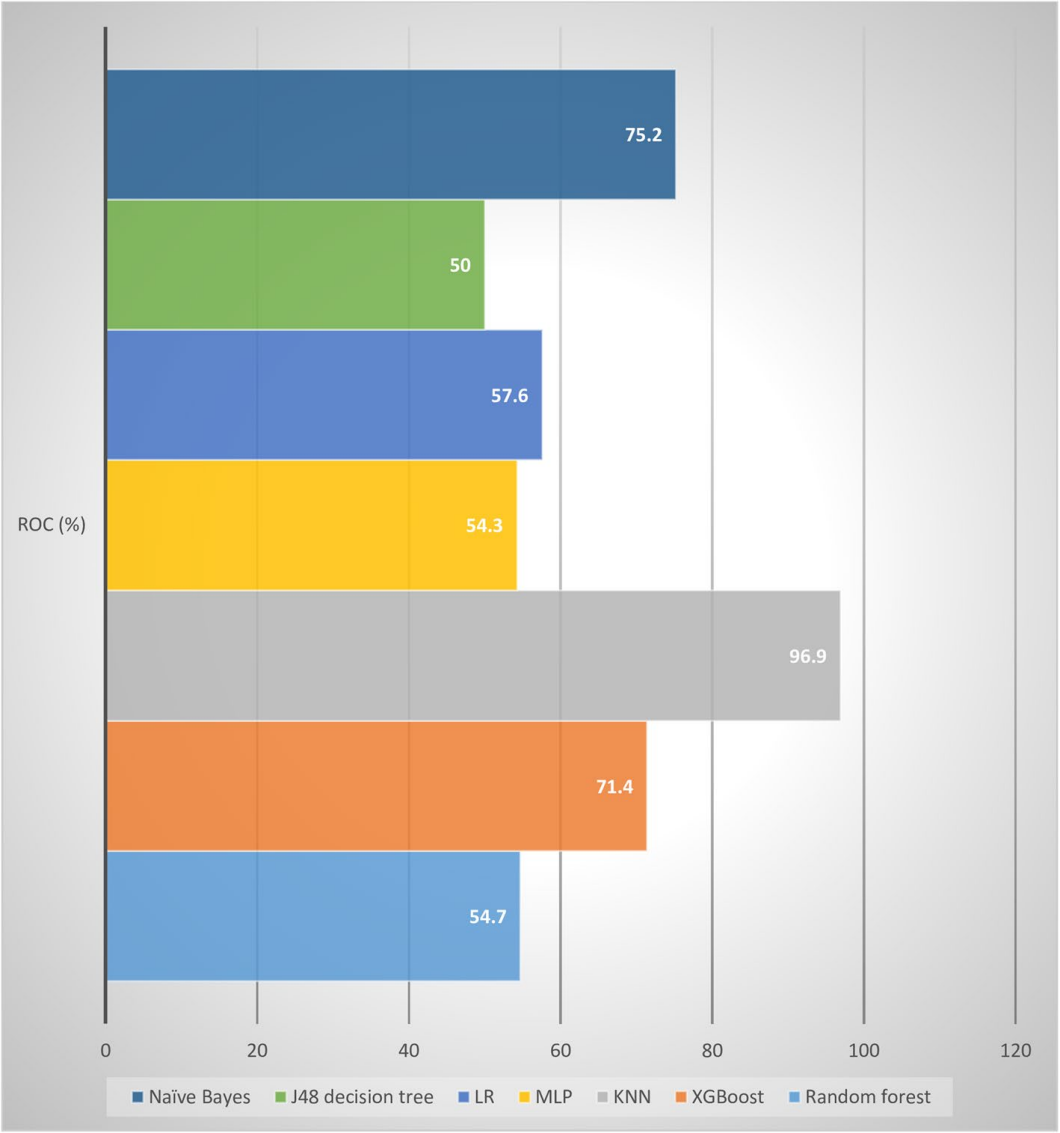
ROC chart of the selected ML algorithm

The experimental findings revealed that the KNN algorithm surpassed other machine learning (ML) algorithms in accurately predicting COVID-19 in-hospital mortality. It achieved impressive performance metrics, including a sensitivity of 95.30%, specificity of 93.30%, accuracy of 95.25%, precision of 92.70%, and an ROC value of 96.90%. Notably, the KNN algorithm utilized a nearest neighbor value of 2, contributing to its success. Figure 6 visually depicts the performance metrics of the ML algorithms used in this study, while Fig. 7 presents a comparison of the area under the ROC curve for these algorithms. According to Fig. 7 the ROC value of KNN was highest (96.9%) compared with the other six algorithm of the study. Remarkably, the J48 algorithm exhibited the lowest performance with an ROC value of 50.0% according to the ROC analysis. For a comprehensive summary of the performance evaluation of each algorithm, please refer to Table 7.

**Table 7:** Performance evaluation of the selected ML algorithms for COVID‑19 death prediction

Algorithm	Sensitivity (%)	Specificity (%)	Precision (%)	Accuracy (%)	F1-secore	ROC (%)
Random forest	99.80	97.60	95.40	95.26	97.55	54.70
XGBoost	95.40	97.60	95.40	95.40	95.40	71.40
KNN	95.30	93.30	92.70	95.26	93.98	96.90
MLP	98.60	98.40	98.60	98.56	98.60	76.20
LR	95.30	93.10	91.00	95.26	93.10	58.40
J48 decision tree	95.40	97.60	95.40	95.40	95.40	50.00
Naïve Bayes	95.30	93.10	91.00	95.26	93.10	75.20

### Association rule result

This study utilized the different feature selection method to select relevant features. Subsequently, the association mining rules were applied using the apriori algorithm for interpretation and a comparison of the best-selected features. From the association mining rules, a total of six most important rules were identified with a confidence value of over 90% and the highest lift or interestingness. The absolute minimum support count of the apriori algorithm was 592 instance with the minimum support value of 0.85 and confidence of 0.9 and the number of cycle performed was 3. The lift value of all rules were above one which is good and the detailed results were presented as follows:

- Rule 1: Chronic liver disease=1 Hematological disease=1 611 ==> pneumonia=1 598, confidence=0.98, lift=1.05, support=3.07
- Rule 2: TB=1 621 ==> Hematological disease=1, 605, confidence=0.97, lift=1.04, support=2.36
- Rule 3: Chronic liver disease=1 615 ==> Hematological diseases=1 598, confidence=0.97, lift=1.04 support=2.21 Rule 4: CLD=1 633 ==> pneumonia=1 615, confidence=0.97, lift=1.05, support=2.35
- Rule 5: TB=1 621 ==> COD=1 599, confidence=0.96, lift=1.04, support=1.9
- Rule 6: Hematologic diseases=1 651 ==> COD=1 627, confidence=0.96, lift=1.04, support=1.83

## Discussion

The objective of this research was to create a machine-learning model capable of accurately predicting the mortality of COVID-19 patients upon their admission to the hospital. What sets this study apart is its emphasis on developing a predictive model using routine laboratory results, therapeutic plans, and demographic characteristics at an early stage, which has not been previously explored. To accomplish this, the researchers analyzed secondary data from hospitals in Ethiopia, granting them access to pertinent patient information such as laboratory results, medical history, patient outcomes, and demographic characteristics.

The researchers conducted a comprehensive study on predicting COVID-19 mortality using various statistical analysis techniques and feature selection methods. They employed machine-learning models such as J48 decision tree, RF, k-NN, MLP, NB, XGBoost, and LR models. Among these techniques, the KNN model exhibited the highest performance with an accuracy of 95.25%. It also demonstrated a sensitivity of 95.30%, precision of 92.7%, specificity of 93.30%, and an ROC of approximately 96.90%. These results indicate that KNN is an exceptionally effective machine-learning technique for this particular task. The study further revealed that the KNN, MLP, Naïve Bayes, and XGBoost models showed good prediction performance, with ROC values above 71.4%. These models also exhibited better diagnostic efficiency compared to other models trained with the same parameters. Overall, this research provides valuable insights into the development of machine learning models for predicting COVID-19 mortality. Implementing these models could potentially enhance patient outcomes and reduce healthcare costs.

Recent studies have investigated the potential of laboratory values in predicting the severity and mortality of COVID-19. Booth et al. developed a prediction model using two machine-learning techniques, Logistic Regression and Support Vector Machines, and identified CRP, BUN, serum calcium, serum albumin, and lactic acid as the top five laboratory values with the highest weights in their model. Their SVM model demonstrated a sensitivity and specificity of 91% and an AUC of 0.93 in predicting mortality [28]. Guan et al. also employed a machine-learning algorithm to retrospectively predict COVID-19 mortality with a sensitivity of 85% [92]. Another scholar developed a machine learning-based predictive model that evaluated binary variables and demonstrated an 87.30% sensitivity and 71.98% specificity in predicting COVID-19 infection [93]. These findings suggest that machine-learning techniques can be useful in predicting COVID-19 outcomes and identifying potential risk factors. This implies that non-invasive methods of mortality are effective for prediction as well as data mining emerged as a policy input.

Several research studies have investigated the application of machine learning (ML) techniques for predicting mortality in patients with COVID-19. One study [30] evaluated the performance of four ML algorithms, namely LR, RF, SVM, and XGBoost. Among these models, the XGBoost algorithm demonstrated the highest performance, achieving an impressive AUC (Area Under the Curve) value of 0.91 [38]. Another retrospective analysis [51] involving 2520 hospitalized COVID-19 patients found that a neural network (NN) model outperformed other models such as LR, SVM, and gradient boosted decision trees, with an impressive AUC value of 0.9760 for predicting patient mortality. These findings underscore the potential of ML techniques, particularly XGBoost and neural networks, in accurately predicting mortality in COVID-19 patients. This implies accuracy of prediction of machine learning importantly implemented for health care service improvement and program design.

In a study involving confirmed COVID-19 patients from five hospitals, researchers developed logistic regression models with L1 regularization (LASSO) and MLP models using local data and combined data. The federated MLP model, with an AUC-ROC of 0.822, outperformed the federated LASSO regression model in predicting COVID-19 related mortality and disease severity [94]. In another study [32], four machine-learning techniques were trained using data from 10,237 patients. Among these techniques, SVM demonstrated the best performance, achieving a sensitivity of 90.7%, specificity of 91.4%, and ROC of 96.3%. Moulaei et al. [39] also predicted the mortality of COVID-19 patients using data mining techniques. They found that Random Forest (RF) was the best model for predicting mortality, with a ROC of 1.00, precision of 99.74%, accuracy of 99.23%, specificity of 99.84%, and sensitivity of 98.25%. Following RF, KNN5, MLP, and J48 were the next best-performing models in predicting mortality. Overall, these studies highlight the effectiveness of various machine-learning models in predicting COVID-19 outcomes, with MLP and RF models showing promising results in predicting mortality and disease severity.

In another study conducted by Moulaei et al. [65], the researchers used machine-learning algorithms to predict the mortality of COVID-19 patients. The results showed that the Random Forest (RF) model performed the best in predicting mortality, with a ROC score of 99.02, precision of 94.23%, accuracy of 95.03%, specificity of 95.10%, and sensitivity of 90.70%. Similarly, Tulu et al. [66] conducted a study involving a cohort of 5,059 patients and found that the Random Forest (RF) model was also the most effective in predicting patient mortality. The area under the curve (AUC) for RF was 0.98, indicating high predictive accuracy. These findings suggest that machine-learning algorithms can be valuable tools in predicting mortality outcomes for COVID-19 patients. In a recent study, predictors of COVID-19 mortality were identified for patients who were admitted with a confirmed diagnosis. The study found that certain factors were more significant in predicting mortality, such as being male, requiring ICU admission, alcohol consumption, smoking, experiencing symptoms such as headache, chills, pneumonia, fever, and receiving oxygen therapy. On the other hand, factors such as TB, COPD, muscular pain, chest pain, confusion, sore throat, cardiac disease, chronic liver disease, cough, hematologic disease, nausea/vomiting, loss of taste, loss of smell, and hypertension were found to be less important in predicting COVID-19 mortality. Other studies have also used machine learning algorithms to identify important predictors of COVID-19 patient mortality. These selected features were then used as inputs to develop machine learning-based models for severity, deterioration, and mortality risk analysis of COVID-19 patients. According to recent research, certain factors have been identified as strong predictors of mortality in COVID-19 patients. Predictors identified in previous literatures were gender [11, 12, 18, 28, 38, 43, 44], low consciousness [11, 17, 18, 41], dry cough [15, 17, 18, 28, 43, 51], fever [12, 17, 18, 42, 43, 48, 49], comorbidity conditions associated with poor prognosis including hypertension [38, 41, 43, 44, 46], lung disease including chronic obstructive lung disease [11, 16, 28, 41], cardiovascular disease [38, 41, 42, 46, 48, 95], pneumonia [12, 17, 44, 49, 95], and chronic renal disease [12, 15, 17, 18]. On the other hand, sore throat [12, 28, 38, 41], myalgia and malaise [12, 38, 46] diarrhea and GI symptoms [42, 48, 51], and headache [12, 17, 49] have the least importance for predicting of mortality. Recent research has identified predictors for COVID-19 patient recovery that differ from those found in previous studies. These predictive features could help healthcare professionals prioritize early intervention, leading to better recovery rates for patients. This approach would not only enhance the quality of healthcare services, but also alleviate the burden on healthcare workers and reduce overall patient care costs.

Machine learning has the potential to greatly benefit clinicians and healthcare providers who are treating patients with COVID-19. By identifying important features early on, proposed algorithms can predict patient mortality with high levels of accuracy, precision, sensitivity, and specificity, as well as an optimum ROC. This prediction can lead to optimal use of hospital resources, particularly for patients with critical conditions, and can help provide better quality care while reducing medical errors due to fatigue and long working hours in the ICU. Valid predictive models can improve the quality of care and increase patient survival rates, by identifying high-risk patients and adopting the most effective assistive and treatment care plans. This approach can help reduce ambiguity, by offering clinicians quantitative, objective, and evidence-based models for risk stratification, prediction, and eventually episode of the care plan. By adopting this approach, clinicians can devise better strategies to reduce complications and improve patient survival rates.

## Conclusion

Our study aimed to develop a new model for predicting the mortality risk of COVID-19 patients using hospital report data from different countries. This model incorporates various factors such as clinical, demographic, risk factors, and therapeutic features. We conducted an extensive review of a large dataset and found that our model has the highest predictive capacity compared to existing literature. The main purpose of this model is to prioritize early treatment for high-risk patients and optimize the use of limited healthcare resources during the ongoing pandemic.

We strongly believe that our proposed technique has the potential to significantly improve decision-making processes in healthcare systems. It can enable precise and targeted medical treatments for COVID-19, empowering medical staff worldwide to effectively triage patients and accurately assess their health and mortality risks. Our study specifically focused on creating and evaluating machine learning-based prediction models for in-hospital mortality, using 23 key clinical predictors. Among the seven machine learning algorithms we tested, the K-nearest neighbors (KNN) model demonstrated the highest classification accuracy. This suggests that our model can effectively predict the mortality risk of hospitalized COVID-19 patients, optimizing the allocation of limited hospital resources.

Importantly, our model can identify high-risk patients as early as the time of admission or during hospitalization. The twenty-three predictors of COVID-19 mortality identified in our predictive model can be considered by policymakers and program designers in the healthcare system. Additionally, the healthcare workforce can pay attention to these predictors when managing COVID-19 patients.

In conclusion, integrating machine learning algorithms with comprehensive hospital databases allows for accurate classification of COVID-19 patient mortality risk. This advancement holds great promise in improving healthcare outcomes and resource management during the ongoing pandemic.

### Limitation and strength

This study was designed as a retrospective analysis, using documented data that were irregular or imbalanced. To address this issue, we took steps to balance the dataset by removing noise and inadequate records. Specifically, we focused on addressing the problem of imbalanced classes, where the number of records related to the deceased class was significantly lower than the recovery or alive class (32 vs 664). To evaluate the performance of each machine learning algorithm, we employed different criteria. Additionally, we conducted external validation of the proposed model using multi-center country-level data, aiming to enhance the generalizability of our predictions. While it would have been beneficial to include features related to lung CT or radiology images, these were not included in our study. We recommend that future researchers consider incorporating these features to further enhance prediction accuracy. It is important to note that our study only considered routine clinical, demographic, and therapeutic features of patients upon admission. We did not have information about the time span from symptom onset to admission, which could have influenced the sampled features. Therefore, it is crucial to monitor the dynamic variations of significant features over time to better identify patients at higher risk of poor outcomes in a timely manner. Furthermore, we excluded patients under the age of 18 and those discharged from the emergency department from our study. Including these individuals may have yielded different results and should be considered in future investigations.

Overall, while our study provides valuable insights using the available data, there are areas for improvement and avenues for further research to enhance the understanding and prediction of outcomes in similar patient populations.

### Supplementary Information

Supplementary Material 1.

## Data Availability

All necessary data included in the manuscript.

## Abbreviations

AI: Artificial Intelligence
COVID-19: Coronavirus disease of 2019
Cr: Creatinine
CRP: C reactive protein
ICU: Intensive care unit
IQR: Interquartile range
LR: Logistics regression
LIME: Local interpretable model-agnostic explanation
LIME-SP: Local interpretable model-agnostic explanation submodular-pick
ML: Machine learning
RF: Random forest
ROC: Receiver operating characteristic curve
RT-PCR: Reverse transcription-polymerase chain reaction
WBC: White blood cells count
