# Supplementary material for: Machine learning algorithms for predicting COVID-19 mortality in Ethiopia
Source: BMC Public Health. 2024 Jun 28;24:1728. doi: 10.1186/s12889-024-19196-0 (PMC11212371; doi:10.1186/s12889-024-19196-0)
Supplement: Supplementary file 1 — Supplementary Material 1. [file 12889_2024_19196_MOESM1_ESM.docx]

Supplementary table 1: Identifying the initial list of features affecting mortality of COVID‑19 patients in Ethiopia

| Class | Number of features suggested | Delphi round score | | Number of final selected features | Included features | Excluded features |
| --- | --- | --- | --- | --- | --- | --- |
|  |  | >=70% | <70% |  |  |  |
| Demographic | 11 | 4 | 7 | 4 | Age, BMI, length of hospitalization, occupation | Residence, educational status, ethnicity, kebele, blood type, income, marital status |
| Risk factors | 19 | 15 | 4 | 15 | ICU admission, DM, hypertension, smoking, alcohol use, HIV/AIDS, TB, Malnutrition, CLD, cancer, CKD, COP, any hematological disease, cardiac disease | Types of contact, prevention practice, recent travel, family history of COVID-19 |
| Clinical manifestation | 24 | 15 | 9 | 15 | Cough, Shortness of birth/dyspnea, sore throat, runny nose, loss of taste, loss of smell, confusion,  muscular pain, chill, fever, cough, nausea/ vomiting, chest pain  headache | Conjunctivitis, weakness, sneezing, pharyngitis, mucus, hemoptysis, anorexia, dry mouth, decrease consciousness |
| Laboratory results | 24 | 11 | 13 | 11 | White blood cell count, platelet count, absolute lymphocyte count, absolute neutrophil count, blood urea nitrogen, glucose, lactate dehydrogenase, alkaline phosphatase, erythrocyte sedimentation rate, C-reactive protein | Aspartate aminotransferase, alanine aminotransferase, hypersensitive troponin, Hematocrit, red cell count, hemoglobin, total bilirubin, thromboplastin time,  prothrombin time, albumin calcium, phosphorus, magnesium |
| Therapeutic results | 1 | 1 | 0 | 1 | Oxygen therapy |  |
| Total | 80 |  | | 46 |  |  |
